# Subcutaneous Eyelid Dirofilariasis Masquerading as an Eyelid Tumor: A Rare Case Report and Literature Review

**DOI:** 10.7759/cureus.70673

**Published:** 2024-10-02

**Authors:** Ayyakutty Muni Raja, Harikrishnan Marappan

**Affiliations:** 1 Ophthalmology, All India Institute of Medical Sciences, Madurai, Madurai, IND; 2 Ophthalmology, Karuna Institute of Medical Sciences, Palakkad, IND

**Keywords:** dirofilariasis, lid tumor, mosquito, parasitic infection, zoonosis

## Abstract

Subcutaneous eyelid dirofilariasis is a rare parasitic infection that can be mistaken for eyelid tumors due to its unusual presentation. In this report, we examine two cases in which patients initially presented with eyelid swellings that were initially diagnosed and treated as preseptal cellulitis in another medical facility. These patients were subsequently referred to our facility to rule out the possibility of a lid tumor. Upon further investigation, both cases were identified as subcutaneous dirofilariasis, a condition caused by the filarial nematode, *Dirofilaria* species. The diagnosis was confirmed through histopathological examination, revealing the presence of the parasite. A comprehensive review of the literature is also provided, highlighting the epidemiology, clinical features, diagnostic challenges, and treatment options for this uncommon condition. The cases underscore the importance of considering parasitic infections in the differential diagnosis of eyelid tumors, especially in endemic areas, to ensure accurate diagnosis and appropriate management.

## Introduction

Ocular dirofilariasis is an uncommon zoonotic nematode type of parasitic infection caused by *Dirofilaria* species that belong to the *Onchocercidae* family [1]. It primarily affects dogs, cats, and foxes more often than humans. Dirofilariasis is most commonly seen in Mediterranean countries due to favorable climatic changes [2]. Out of 40 species of the *Dirofilaria* family, *D. repens, D. immitis, D. tenius, D. ursi, *and* D. spectans* are common subtypes affecting humans [3]. Dirofilariasis commonly affects the pulmonary, subcutaneous, and ocular areas in humans. Humans are the final host of dirofilariasis, which affects humans through mosquito vectors like *Anopheles*, *Aedes*, and *Culex* mosquitos. Subcutaneous dirofilariasis is a significantly underreported global disease. The worldwide reported cases total only 800, with 73 cases documented in India since 1977. Furthermore, occurrences of ocular involvement are exceptionally rare [4]. Ocular dirofilariasis typically involves the periorbital region, subconjunctival space, and vitreous cavity areas.

## Case presentation

Case 1

**Figure 1:**
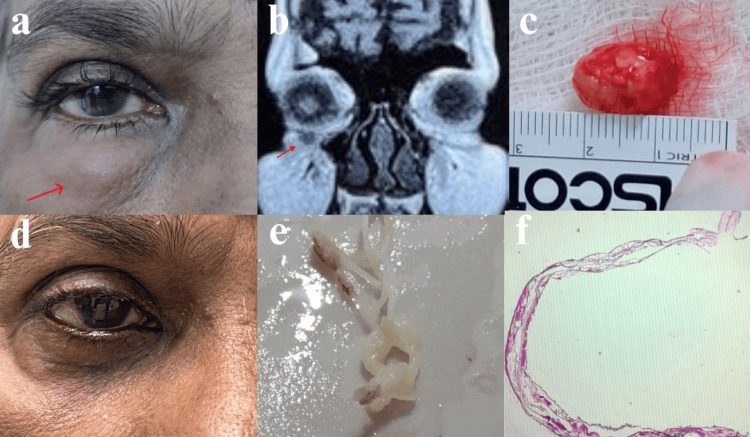
Case 1: a) preoperative, b) CT orbits screening, c) intraoperative, d) postoperative, e) macroscopic, f) microscopic Figure a: Diffuse, irregularly shaped soft swelling measuring approximately 1.5 × 1 cm was observed, extending from the medial canthal area to the middle of the lower lid and 1.5 cm below the lower lid margin of the right eye. Figure b: CT orbits screening showed ill-defined soft tissue thickening of the right lower palpebrae in the preseptal compartment of the right eye lower eyelid, suggestive of inflammatory etiology. Figure c: Excised specimen of grayish-white nodular soft tissue lesion measuring 1.5 × 1 × 0.2 cm. Figure d: postoperative. Figure e: Macroscopic cut section showing two worms, the largest one measuring 4.5 cm and the smallest worm measuring 3 cm in length. Figure f: Microscopic appearance showing fragments of parasite displaying cuticle with longitudinal ridges and muscle layer.

A 65-year-old female presented with persistent swelling in the lower lid of her right eye over the past year. She had previously been diagnosed with preseptal cellulitis and received treatment with systemic antibiotics. However, the swelling did not improve, prompting her to seek further evaluation and management from the Department of Ophthalmology. Upon examination, a diffuse, irregularly shaped soft swelling measuring approximately 1.5 × 1 cm was observed, extending from the medial canthal area to the middle of the lower lid and 1.5 cm below the lower lid margin of the right eye (Figure 1a). No abnormalities were identified in both the anterior and posterior segment examinations. Due to a suspected lower lid tumor, a CT orbits was performed, which showed ill-defined soft tissue thickening of the right lower palpebrae in the preseptal compartment of the right eye lower eyelid - suggestive of inflammatory etiology (Figure 1b). Following routine blood investigations (Table 1), the patient underwent an excision biopsy, and the specimen was sent for histopathological analysis. During the procedure, a skin incision was made over the swelling, and the nodule was dissected from the surrounding structures and excised. Hemostasis was achieved, and the wound was closed in layers using absorbable sutures. Histopathological examination revealed features consistent with dirofilarial infestation, showing fragments of the parasite with a cuticle, longitudinal ridges, muscle layer, and body cavity surrounded by dense acute and chronic inflammation in fibroconnective tissue (Figure 1c, 1d, 1e, 1f). The patient's postoperative recovery has been remarkably positive, with subsequent evaluations at 15 days and one month following the surgical procedure showing continued progress.

Case 2

**Figure 2:**
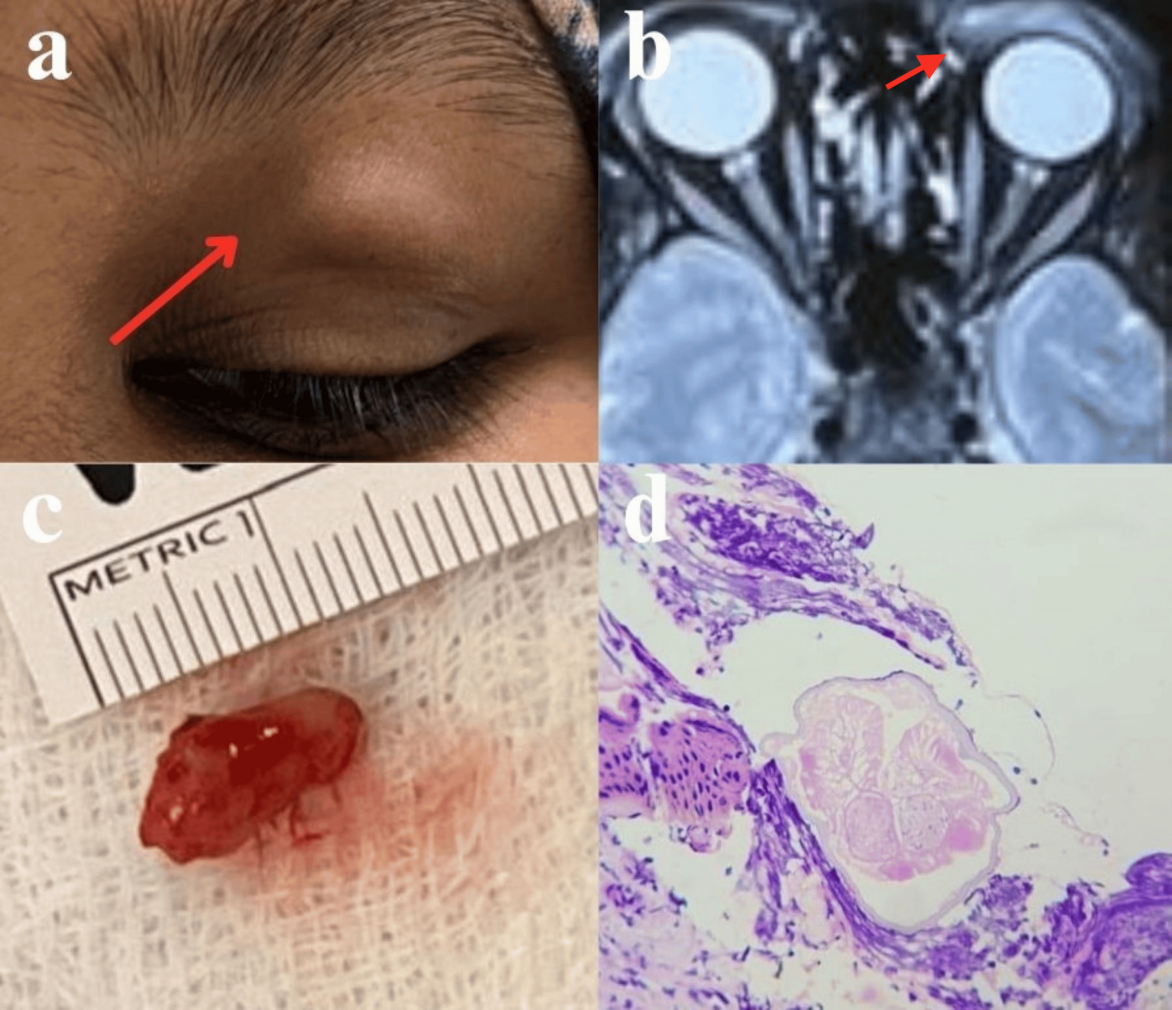
Case 2: a) preoperative, b) CT orbits and paranasal sinus (PNS) screening, c) macroscopic, d) microscopic Figure a: Soft, irregularly shaped swelling measuring 1 × 0.5 cm was observed in the middle of the left eye  upper eyelid, 1 cm below the supraorbital region, and 2.5 cm above the lid margin. Figure b: CT orbits and paranasal sinus (PNS) screening showed ill-defined thickening of preseptal soft tissue of the left eye upper eyelid, suggestive of inflammatory etiology. Figure c: Excised specimen of small grayish-white nodular mass measuring 1 × 0.5 × 0.5 cm. Figure d: Microscopic appearance shows a parasitic worm having thin acellular outer cuticle, inner radiating muscle layer, and innermost double uterus.

A 40-year-old woman complained of painless swelling in her left upper eyelid for the past year. Upon examination, a soft, irregularly shaped swelling measuring 1 × 0.5 cm was observed in the middle of the upper eyelid, 1 cm below the supraorbital region, and 2.5 cm above the lid margin (Figure 2a). The examination of the anterior and posterior segments revealed normal results, with a suspicion of a parasitic or foreign body granuloma. Subsequently, a CT orbit was conducted to investigate further and exclude the presence of a foreign body or tumor, which showed ill-defined thickening of the preseptal soft tissue of the left eye's upper eyelid, suggestive of inflammatory etiology (Figure 2b). Following routine blood investigations, the patient underwent an excision biopsy and histopathological examination (Table 1). The procedure entailed making a skin incision over the swelling, excising the nodule, and dissecting it from the surrounding structures after local infiltration under aseptic precautions. The excised specimen was dispatched for histopathological analysis. The histopathological examination revealed fibrocollagenous tissue with extensive lymphoplasmacytic and moderate eosinophilic infiltrates and ill-circumscribed, variably sized collections of epithelioid histiocytes and occasional Langhans giant cells. In addition, thin refractive eosinophilic fragments of parasitic cuticle were observed, along with a cross-section of a parasitic worm displaying a thin acellular outer cuticle, inner radiating muscle layer, and innermost double uterus (Figure 2c, 2d). The postoperative recovery exhibited positive progress, and the patient underwent subsequent reviews at 15 days and one month following the surgical procedure.

**Table 1:** Routine blood investigation reports of both patients showing normal results except raised erythrocyte sedimentation rate (ESR) suggestive of inflammatory cause

Parameter	Case 1	Case 2	Normal range
Hemoglobin (H )	14 gm/dl	8.2 gm/dl	12 to 15 gm/dl
Random blood sugar (RBS)	96 mg/dl	85 mg/dl	7-140 mg/dl
HIV 1 and 2 antibodies	0.12 (nonreactive)	0.14 (nonreactive)	<0.90 (nonreactive)
Hepatitis B surface antigen (HBSAG)	0.20 (nonreactive)	0.26 (nonreactive)	<0.90 (nonreactive)
Erythrocyte sedimentation rate ( ESR )	36 mm/hour	43 mm/hour	0-20 mm/hour (female)
Bleeding time (BT)	2 minutes 10 seconds	2 minutes 20 seconds	2-7 minutes
Clotting time (CT)	4 minutes 15 seconds	4 minutes	3 – 10 minutes

## Discussion

Ocular dirofilariasis is a zoonotic filariasis caused by a nematode worm called *Dilofilaria*. The disease is transmitted to humans through the bite of *Aedes*, *Culex*, or *Anopheles* mosquitoes. Dogs are the main carriers of the infection, and humans are incidental hosts. Human infection can be caused by *D. immitis*, *D. tenius*, *D. ursi*, and *D.* *repens*. Among these, the condition associated with *D. repens* is the most common and widely distributed form of dirofilariasis with medical significance worldwide [5]. In India, dirofilariasis is predominantly documented in published literature from southern regions, specifically in Kerala, Karnataka, and Tamil Nadu [6].

Ocular dirofilariasis can be seen in various parts of the eye, including the subconjunctival space, eyelids, intraorbital area, and anterior chamber, and occasionally spreads into the vitreous cavities [7]. Among the 40 subtypes of the *Dirofilaria* family, *D. repens*, *D. immitis*, *D.* *tenuis*, *D. ursi*, and *D. spectans* are common subtypes responsible for human infections. *D. repens* is most commonly responsible for subcutaneous ocular infection, while *D. immitis* typically causes pulmonary dirofilariasis [8,9]. The clinical manifestations of ocular dirofilariasis can vary widely depending on the location and number of larvae. Redness, pain, swelling, and foreign body sensation in the eye are common symptoms. In our cases, swelling and pain were the main symptoms. More severe complications can include vision loss, retinopathy, and uveitis [10]. Ocular dirofilariasis diagnosis is based on clinical examination, imaging studies, and laboratory tests. Imaging studies such as ultrasound, MRI, or CT help identify parasites within the ocular system [11]. Blood tests often reveal increased numbers of eosinophils in most of the infections. In our cases, except for the raised erythrocyte sedimentation rate (ESR), other blood investigations are within normal limits. A definitive diagnosis can be confirmed through histopathological examination following surgical parasite removal. The characteristic features of *D. repens* were the short cephalic space, longitudinal thick cuticular ridges along the body, and large muscle cells, as evident in our cases [11].

Anti-helminthic drugs are not indicated for ocular dirofilariasis, as the parasites are reproductively inactive and do not cause filaremia like other filarial worms. Surgical removal of the worm is the definite treatment, and there is no role for postoperative anti-helminthic treatment [12].

Alteration of climate conditions may potentially lead to an escalation in the prevalence of analogous zoonotic infections. Such variables can exert an influence on mosquito breeding and disease transmission. Elevated temperatures may curtail the lifespan and generation time of diverse vector species, rendering them more conducive to the transmission of pathogenic agents. Climate variations can also impact land use patterns, thereby influencing the distribution of vector species' habitats and facilitating the dissemination of diseases to novel regions and larger populations [13].

Mosquito-borne diseases, particularly those caused by *Dirofilaria* species, pose significant public health challenges, often leading to severe conditions such as dirofilariasis in both humans and animals. Early diagnosis is crucial for effective management and treatment, and a clinical algorithm can aid in prompt identification. This algorithm should begin with a detailed patient history focusing on potential exposure to mosquito-prone areas, followed by a thorough physical examination for signs like subcutaneous nodules, respiratory symptoms, or ocular involvement. If suspected, diagnostic steps should include blood tests for eosinophilia, imaging studies to locate migrating parasites, and specific serological tests or molecular techniques to confirm *Dirofilaria* infection.

Preventing dirofilariasis primarily involves the avoidance of mosquito bites. To mitigate potential public health concerns, a combination therapy of ivermectin and doxycycline can be administered to eradicate *Dirofilaria* infection in dogs. This approach serves as a proactive measure to address public health implications associated with the infection [14].

Multidisciplinary collaboration among entomologists, parasitologists, veterinarians, clinicians, and public health experts is essential to address the public health implications of *D. repens* infection. This collaborative effort is of great relevance and significance.

## Conclusions

Dirofilariasis should be considered when diagnosing inflammatory and non-inflammatory mass lesions in the periorbital tissues. While ocular dirofilariasis is commonly found in Mediterranean countries, recent cases have been documented in our region, underscoring the emergence of this zoonotic disease. Global climate change may have significantly impacted the distribution and infection rates of dirofilariasis as a vector-borne disease. Therefore, clinicians should bear in mind the potential for this zoonotic infection when they come across localized nodules in the periorbital region of the eye.
